# The Kidneys and the Cardiovascular System

**Published:** 1901-11-23

**Authors:** 


### Hospital Clinics and Medical Progress.

### THE KIDNEYS AND THE CARDIO-VASCULAR SYSTEM.

In this month's *Practitioner* Sir William Broadbent discusses the effects of renal disease upon the circulation. First comes increased resistance in the arterio-capillary network; then there is a contraction of the small arteries—the stop-cock action of George Johnson; then the heart "rises to the occasion" and contracts with greater vigour, so that on the arterial side of the circulation we have a resistance in front and increased pressure behind, and consequently a high pressure in the intervening arteries from the aorta onwards. Naturally the first direct effect of this, if the vessels were flaccid tubes, would be merely to stretch them. In fact, however, it is otherwise. Just as the heart was "equal to the occasion" so are the blood vessels. A "physiological increase in the muscular fibres" takes place so that the arteries may still be able to control the circulation, dilating or contracting as may be required, even in the face of the increased pressure. Fibroid changes also take place, so that the arteries become thicker. Ultimately, however, some amount of stretching takes place, and then we have the characteristic "renal pulse" in its full development—large, the pulse wave long and dwelling on the finger, gradual in its rise and fall, or more sudden if the arterial system has undergone much degeneration; the artery full between the beats, and thick walled, rolls under the finger like another tendon at the wrist, is followed with ease half way up the forearm, and is usually tortuous. As with the vessels so with the heart. At first increased vigour, with marked apex-thrust and accentuation of the aortic second sound. Gradually hypertrophy, displacement of the apex downwards and slightly outwards, with its action showing a general heave as well as a distinct apex-push. Then fibroid changes and degeneration; and it may often seem to depend upon some quasi-accidental circumstance whether cerebral arteries shall be ruptured by the pressure which the heart is able to maintain, or the heart shall be beaten and fail to hold up the blood current against the obstructions which have arisen in its path. Meanwhile as the changes in the heart and blood vessels advance so do the changes in the kidney; hence the symptoms are partly due to the one condition, partly to the other. Thus we have headache, troubles of the digestion, loss of flesh, breathlessness, sleeplessness, irregular action of the heart, and in later stages, perhaps, attacks of nocturnal dyspnœa. So much for cases of contracted granular kidney. The effects of tubular nephritis on the circulation are not so easily followed, for other factors enter into the problem, a toxic influence acting directly upon the heart muscle in addition to the mere mechanical influence of increased blood pressure. The tendency to obstruction in the capillaries from the presence in the blood of nitrogenous impurities, is, however, always present, and the difference in the results largely arises from the fact that in this disease the heart fails to respond to the obstruction before it, and instead of overcoming it by hypertrophy becomes dilated. "The obstruction has developed too rapidly for the heart to keep pace with it, and the heart has been at the same time subjected to debilitating influences." The pulse then remains flaccid, and the heart weak; there is no effort on the part of the constitution towards recovery; and it is only when the pulse becomes firm and long that the amount of urine increases, the proportion of albumin diminishes and the first step towards recovery is taken. So far as the proper functionisation of the organs is concerned, the maintenance of a certain degree of arterial tension—a tension, in fact, proportional to the resistance—is essential. On the other hand, the maintenance of such a tension is exhausting to the heart and dangerous to the arteries, leading on the one hand to dilatation and heart failure, and on the other to rupture of arteries and cerebral hæmorrhage. So far, then, as the high tension is concerned the first therapeutic indication is immediate and free venæsection on the occurrence of uræmic convulsions. Sixteen or twenty ounces of blood should be taken at once, followed by a calomel purge. If a single withdrawal of blood do not stop the convulsions it may be repeated, and recurrent uræmic convulsions may be met by further venæsection. It must further be remembered that the damage to the vessels and heart, by which much of the suffering attending renal disease is brought about, is due to high arterial tension, the reduction of which ought therefore to be an object continually held in view. For this purpose the vascular relaxants, such as nitro-glycerine, the nitrites and the tetra-nitrite of erythrol may be employed. Unfortunately their action is evanescent. The best means of exercising a definite influence on unduly high intra-arterial pressure is, according to Sir William Broadbent, through mercurial aperients. A dose of calomel, three to five grains, will often avert impending convulsions or prevent their recurrence, will relieve headache, stupor, and twitchings, and may prevent uræmic paroxysmal dyspnœa in advanced kidney disease. So also a single grain of hydrarg. c. cret., with rhubarb, or colocynth and hyoscyamus, once, twice, or three times a week according to the degree of tension in the pulse, exercises a favourable influence in the early stages of chronic Bright's disease, both on the symptoms and on the progress of the disease.

